# Immunoproteasome inhibition triggers protein stress and apoptosis in cells of B cell lineage without impairing vaccination-induced antibody responses

**DOI:** 10.1038/s41420-025-02818-w

**Published:** 2025-11-24

**Authors:** Dennis Mink, Franziska Oliveri, Julia Otto, Nazlim Kutsi, Carolina Gonzalez Siebold, Tony Muchamuel, Jun Li, Michael Basler

**Affiliations:** 1 Institute of Cell Biology and Immunology Thurgau (BITG) at the University of Konstanz, Kreuzlingen, Switzerland; 2 Division of Immunology, Department of Biology, University of Konstanz, Konstanz, Germany; 3 Department of Research, Kezar Life Sciences, South San Francisco, CA USA; 4 Department of Urological Oncology Surgery, Chongqing University Cancer Hospital, Chongqing, China

**Keywords:** Immunology, Cell biology

## Abstract

The immunoproteasome (IP) is a specialized form of the 26S proteasome, in which the catalytic subunits β1c, β2c, and β5c of the standard proteasome are replaced by LMP2, MECL-1, and LMP7. The IP is constitutively expressed in hematopoietic cells and its expression in non-hematopoietic cells can be induced by IFN-γ. The IP plays a crucial role in different immune functions, including MHC class-I ligand generation, cytokine production, and T helper cell differentiation. Selective inhibition of the IP has shown therapeutic benefits in treating different autoimmune diseases in pre-clinical animal models. However, the effect of IP inhibition on antibody production in viral infection and vaccination has remained underexplored. In this study, we used ONX 0914, an LMP7/LMP2-selective inhibitor of the IP, to study the effect of IP inhibition on B cells and antibody production. In vitro, continuous exposure to ONX 0914 in a human B cell lymphoma cell line and primary murine B and plasma cells led to poly-ubiquitinated protein accumulation, increased apoptosis, reduced antibody secretion, and impaired immunoglobulin class-switch. However, induction of virus neutralizing antibodies was not affected in IP inhibitor-treated mice. Furthermore, IP inhibition neither impaired vaccine-induced antibody responses, nor affected different B cell populations in two different vaccination models. These findings suggest that IP inhibition does not compromise vaccination efficacy and anti-viral humoral immunity, supporting the potential of IP-targeted therapies for autoimmune diseases.

## Introduction

The proteasome is the central proteolytic system in cells, regulating the degradation of proteins into peptides, which can enter the MHC class-I restricted antigen presentation pathway to be presented to cytotoxic T cells [1, 2]. The 20S core particle of the 26S proteasome contains two inner β-rings harboring three different catalytic subunits: β1, β2, and β5. These subunits are responsible for caspase-like, trypsin-like, and chymotrypsin-like activities, respectively [3, 4].

The immunoproteasome (IP) is a specialized variant of the proteasome that replaces these catalytic subunits of the standard proteasome with the inducible subunits LMP2, MECL-1, and LMP7. While the IP is predominantly expressed in immune cells, its expression is also upregulated in response to the pro-inflammatory cytokine IFN-γ in non-immune cells [5–7]. Interestingly, due to its strong association with inflammation, immunoproteasome subunits have been proposed to predict the efficacy of immunotherapy in muscle-invasive bladder cancer [8]. Many studies have demonstrated the importance of the IP in immune functions such as antigen processing for MHC class I presentation, T cell activation and differentiation, as well as cytokine production [7, 9–14]. Due to its prominent role in regulating immune processes, the IP has emerged as a potential therapeutic target in autoimmune diseases. IP-selective inhibitors, such as the LMP7/LMP2-selective epoxyketone inhibitor ONX 0914, have shown promising therapeutic effects in several experimental models for autoimmunity [13, 15–20]. In addition, Zetomipzomib [21] a selective inhibitor of the immunoproteasome, is currently being investigated in clinical trials for the treatment of autoimmune diseases, including dermatomyositis/polymyositis, systemic lupus erythematosus, lupus nephritis and autoimmune hepatitis.

Cells of the B cell lineage are central to humoral immunity, being the sole producers of immunoglobulins, either expressed on the surface as B cell receptors or released from short-lived plasmablasts or long-lived plasma cells as secreted antibodies [22, 23]. Naïve B cells usually express membrane-bound IgM and IgD to recognize foreign antigen [24]. Upon encountering an antigen and receiving T cell help, B cells proliferate and differentiate into plasma cells, which produce large amounts of soluble antibodies [25, 26]. Plasma cells heavily depend on an efficient proteasome system to manage the protein-folding and degradation demands associated with high levels of antibody synthesis [27–29]. Previous studies have shown that proteasome inhibition induces the unfolded protein response (UPR) leading to an accumulation of polyubiquitinated proteins, thereby inducing cell death in highly secretory cell types such as plasma cells [30–32]. This is evidenced by the clinical effectiveness of the non-selective proteasome inhibitors bortezomib or carfilzomib, which are used as therapeutic agents for treatment of multiple myeloma, a plasma cell malignancy [33, 34]. Due to severe side effects of dual proteasome inhibition, selective inhibition of IP-subunits has been investigated as a therapeutic approach for autoimmune diseases in recent years, including diseases that are mediated by production of autoantibodies, such as systemic lupus erythematosus [35, 36]. Additionally, specific inhibition of the IP has been shown to reduce antibody-mediated allograft rejection in rats, further suggesting an influence of IP inhibition on antibody-secreting cells (ASCs) [37]. Despite these findings, the precise effects of IP inhibition on B cells and plasma cells, particularly in relation to antibody responses after vaccination, remain incompletely understood.

Using an IP-inducible human lymphoblastoid cell line, along with primary cells from both mice and humans, as well as in vivo vaccination models, we aimed to investigate the effects of IP inhibition on B cells and plasma cells. Our findings reveal that continuous treatment with ONX 0914 induces poly-ubiquitin accumulation and activates the UPR in vitro, leading to increased cell death and diminished antibody secretion. Furthermore, primary mouse splenocytes exhibited a reduced class switch from IgM to IgG or IgE when treated with ONX 0914 in vitro. In contrast, in vivo treatment did not affect antigen-specific antibody titers after vaccination or total numbers of different B cell-lineage populations in the spleen and bone marrow, suggesting the presence of in vivo mechanisms reducing the effects of IP inhibition. Despite the susceptibility of B cells and plasma cells to IP inhibition in vitro, vaccination responses remain unaffected. This highlights the potential for IP-targeted therapies in autoimmune diseases without impairing vaccine effectiveness.

## Results

### Immunoproteasome inhibition in a human B cell line induces the UPR, increases cell death and reduces antibody secretion

**Fig. 1:**
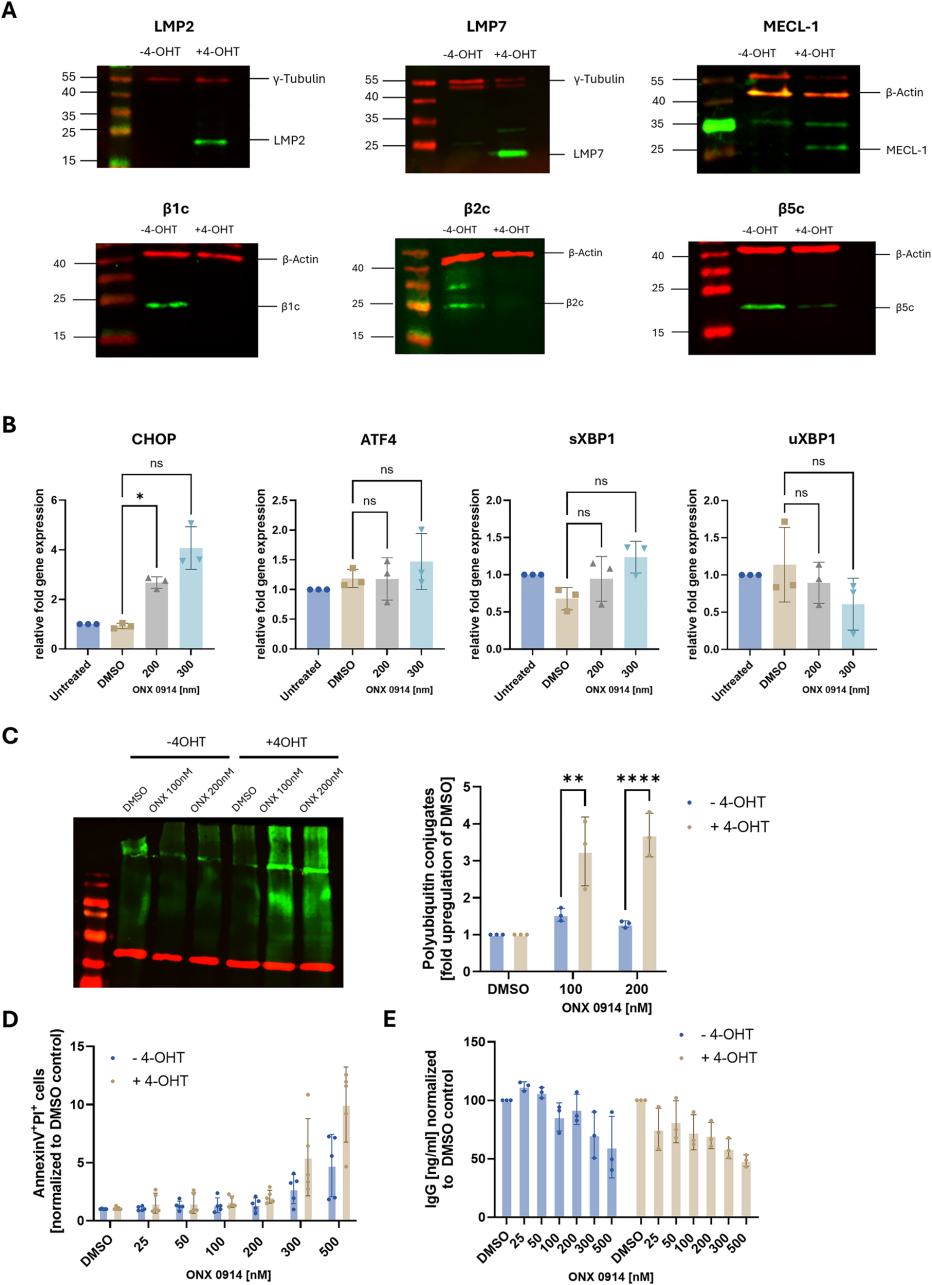
Immunoproteasome inhibition in a B cell lymphoma cell line induces the UPR, increases cell death and reduces antibody secretion. **A** Immunoproteasome expression was induced in LCL721-G27 cells using 4-OHT or left untreated and lysed on day 7 post induction. Lysates were analyzed for LMP7, LMP2, MECL-1, β5c, β2c and β1c by immunoblotting. Quantification of band intensities of proteasome subunits were normalized to loading controls γ-tubulin or β-actin. Uncropped Western Blots are shown in Supplementary Fig. 8. **B** 4-OHT induced LCL721-G27 were treated for 16 h with ONX 0914. Data were normalized to RPL13a. The relative expression of the indicated genes analyzed by real-time RT-PCR compared to untreated cells is shown. Data shows mean ± SD of normalized values derived from 3- independent experiments (*n* = 3). **C** 4-OHT induced and uninduced LCL721-G27 cells were treated for 6 h with ONX 0914 or DMSO and accumulation of ubiquitin conjugates was analyzed by western blot. For quantification, band intensities were normalized to β-actin. Fold upregulation compared to DMSO control is shown as mean ± SD derived from 3 independent experiments (*n* = 3). A two-way ANOVA was performed to determine statistical significance. Uncropped Western Blots are shown in Supplementary Fig. 8. **D**, **E** 4-OHT induced or uninduced LCL721-G27 cells were treated for 24 h with ONX 0914 or DMSO. **D** Apoptosis was analyzed with flow cytometry via Annexin V and PI staining. **E** Supernatant was analyzed for IgG secretion via ELISA. Pooled data from 5 (**D**) or 3 (**E**) independent experiments are presented as means ± SD. Statistics: one-way ANOVA or multiple *t*-tests *P < 0.05; **P < 0.01,**** *P* < 0.0001.

We employed the IP-inducible lymphoblastoid cell line LCL721-G27 as a model to examine the effects of IP inhibition on B cells. LCL721-G27 cells, which lack endogenous LMP2 and LMP7, were stably transfected with a tamoxifen (4-OHT)-inducible system to express these two IP subunits, making it a suitable model for studying the consequences of IP inhibition (Supplementary Fig. 1). We first confirmed successful induction of the IP by performing Western Blot analysis. Treatment with 4-OHT resulted in a drastic upregulation of IP subunits LMP2, LMP7, and MECL-1, whereas the expression levels of the standard subunits β1c, β2c, and β5c were reduced (Fig. 1A). Of note, incorporation of MECL-1 requires expression of LMP7 and LMP2 [38]. Hence, MECL-1 is only incorporated into IPs after induction of LMP2 and LMP7 in these cells. Due to their high antibody production, antibody-secreting cells are sensitive to proteasome inhibition [39]. To evaluate UPR activation after IP inhibition in our system, 4-OHT-induced cells were continuously treated for 16 h with ONX 0914, and the expression of UPR-associated genes was assessed by quantitative RT-PCR. Compared to dimethyl sulfoxide (DMSO)-treated and untreated controls, ONX 0914-treated cells exhibited marked upregulation of CHOP, a key mediator of endoplasmic reticulum (ER) stress-induced apoptosis, while ATF4 expression remained unchanged. Moreover, we observed a tendency for an increase in the spliced form of XBP1 (sXBP1), accompanied by a tendency for a decrease in the unspliced form (uXBP1) (Fig. 1B). These results indicate that ONX 0914 activates the UPR in LCL721-G27 cells. To confirm the presence of ER-stress on protein level, accumulation of poly-ubiquitinated proteins was assessed via western blot. To this effect, LCL721-G27 cells, either induced with 4-OHT (immunoproteasome expression) or left uninduced (standard proteasome expression), were continuously treated with ONX 0914 (or DMSO as control) for 6 h before lysis. Consistent with an activation of the UPR on mRNA level, cells expressing IPs showed a massive increase in the amount of poly-ubiquitinated proteins, whereas no accumulation was observed in uninduced cells (Fig. 1C). Finally, to assess whether the activation of the UPR and the accumulation of poly-ubiquitinated proteins via IP inhibition leads to apoptosis induction, cells were continuously treated for 24 h with ONX 0914 and analyzed for apoptosis induction by Annexin V and PI staining using flow cytometry. Cells induced with 4-OHT showed an increase in Annexin V-PI double-positive cells when treated with ≥300 nM ONX 0914. However, relative to uninduced controls, this difference did not reach statistical significance (Fig. 1D). Consistently, IgG antibodies detected in the supernatant of LCL721-G27 cells via ELISA were similarly reduced after continuous ONX 0914 treatment (Fig. 1E). Collectively, these results indicate triggering of ER stress in IP expressing LCL721-G27 cells after IP inhibition with ONX 0914, resulting in activation of UPR signaling pathways. This ultimately leads to apoptotic cell death, manifesting in reduced antibody secretion.

### Immunoproteasome inhibition of activated primary mouse splenocytes and human PBMCs leads to a reduction in cells of the B cell lineage and reduced IgM and IgG antibody secretion

**Fig. 2:**
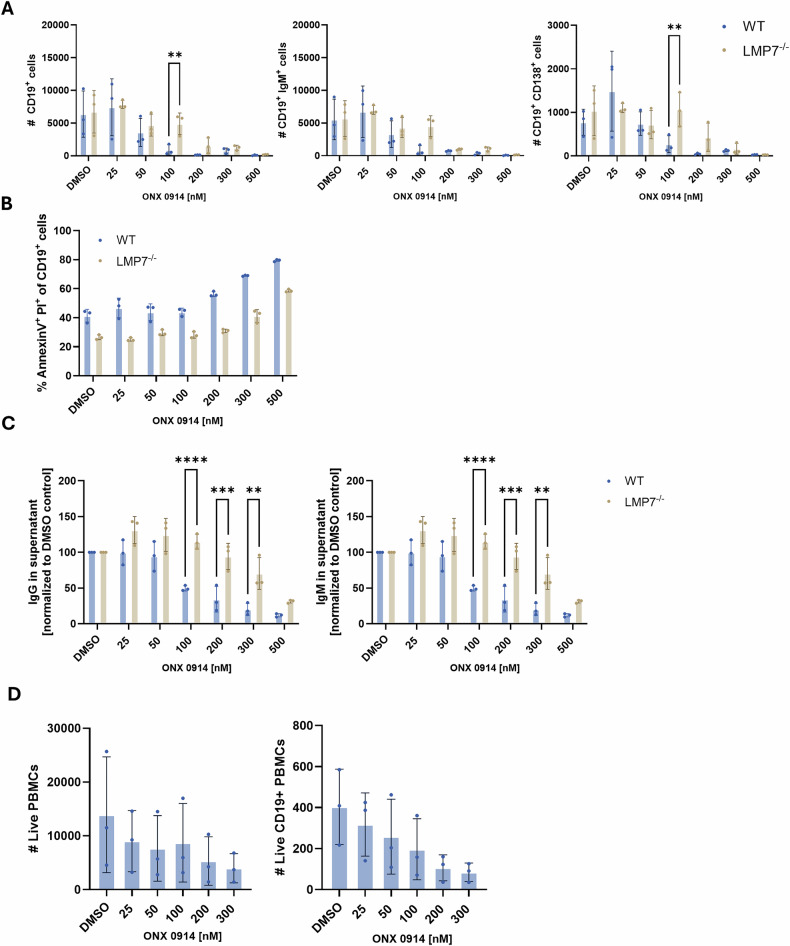
Immunoproteasome inhibition of activated primary mouse splenocytes and human PBMCs leads to reduction in cells of the B cell lineage and reduced IgM and IgG antibody secretion. **A**, **B** C57BL/6 WT or LMP7-deficient mouse splenocytes were activated with 10 µg/ml LPS and treated with different concentrations of ONX 0914 for 72 h. **A** Cells were analyzed via flow cytometry. Cells were pre-gated on live cells and absolute counts of CD19^+^ cells, CD19^+^ IgM^+^ cells and CD19^+^ CD138^+^ cells were assessed. **B** 24 h after treatment with LPS and ONX 0914, cells were harvested and analyzed for AnnexinV^+^ PI^+^ of CD19^+^ cells via flow cytometry. **C** Supernatant was collected for analysis of IgM and IgG antibody secretion via ELISA. **D** Human PBMCs were activated with 1 µg/ml LPS and co-treated with increasing concentrations of ONX 0914. After 72 h, cells were analyzed via flow cytometry. Absolute counts of live cells and live CD19^+^ cells was assessed. **A**–**C** Data from 3 biological replicates is presented as means ± SD. Statistics: two-way ANOVA or multiple *t*-tests **P < 0.01; ***P < 0.001; ****P < 0.0001.

We next addressed the effect of IP inhibition on primary cells of the B cell lineage from mice or humans. Therefore, splenocytes from wild type (WT) C57BL/6 mice or LMP7^–/–^ mice were activated with lipopolysaccharide (LPS) in the presence of increasing concentrations of ONX 0914. Following stimulation for 72 h, cells were stained for viability, as well as the B cell lineage markers CD19, IgM, and CD138, and analyzed via flow cytometry (gating shown in Supplementary Fig. 2). Additionally, supernatant was collected for analysis of IgM and IgG secretion via ELISA. Flow cytometric analysis revealed a decrease in the absolute count of live CD19^+^ cells in WT splenocytes by ONX 0914 treatment in a dose-dependent manner. A similar reduction was observed in CD19^+^, IgM^+^ cells, which correspond to naïve splenic B cells. In LMP7^–/–^ mice, loss of LMP7 is compensated by incorporation of the standard subunit β5c into the proteasome [5, 40]. Additionally, due to interdependent incorporation of proteasome subunits, expression of LMP2 and MECL-1 is also strongly reduced in these mice [41]. Hence, these cells barely express IPs. Cells from LMP7^–/–^ mice only started to show a reduction in different B cell populations at higher concentrations of ONX 0914, demonstrating an IP inhibition-specific effect in wild type cells, while also indicating nonspecific inhibition of standard proteasome subunits at higher concentrations of ONX 0914 (above 100 nM) (Fig. 2A). When cells were left without LPS activation, only very low numbers of live cells were detected after 72 h (Supplementary Fig. 3A). Notably, a population of CD19^+^CD138^+^ cells arose after 72 h LPS treatment, which was present only in minimal amounts in LPS-untreated cells (Supplementary Fig. 3B). This population could represent plasmablasts, which may have differentiated from naïve B cells due to LPS stimulation. Similarly, this population was drastically reduced through ONX 0914 treatment in WT cells, and only at higher concentrations in LMP7^–/–^ cells (Fig. 2A). To determine whether the reduced numbers of cells were due to cell death or an inhibition of LPS-induced proliferation, we additionally stained LPS-treated cells for Annexin V and PI, 24 h post stimulation. The dose-dependent increase of CD19^+^ Annexin V-PI double positive cells after ONX 0914 treatment in WT cells heavily implies induction of apoptosis through prolonged ONX 0914 treatment (Fig. 2B). ELISA analysis of culture supernatants showed that activated splenocytes derived from both WT mice and LMP7^–/–^ mice secreted high levels of IgM and IgG compared to unstimulated cells (Supplementary Fig. 3C). The amount of both IgM and IgG was dose-dependently reduced in supernatants from WT cells, while LMP7^–/–^ cells were only affected at higher concentrations of ONX 0914 (Fig. 2C). To further strengthen our data, human peripheral blood mononuclear (PBMCs) were similarly activated with 1 µg/ml LPS for 72 h in the continuous presence of ONX 0914. Consistent with the data from mouse splenocytes, treatment with ONX 0914 markedly reduced the numbers of CD19^+^ PBMCs (Fig. 2D). These results show that ONX 0914 treatment can induce apoptosis in primary activated B cells of both murine and human origin, providing further evidence for the susceptibility of B cells and ASCs to IP inhibition. The differential sensitivity between WT and LMP7^–/–^ cells supports an IP-specific effect at lower inhibitor concentrations and suggests that at higher concentrations, off-target effects on the standard proteasome may occur. However, since B cells mainly express IPs [7], inhibition of standard proteasomes seems not to be relevant in these cells.

### Immunoproteasome inhibition suppresses class-switch of primary mouse CD19^+^ cells in vitro

**Fig. 3:**
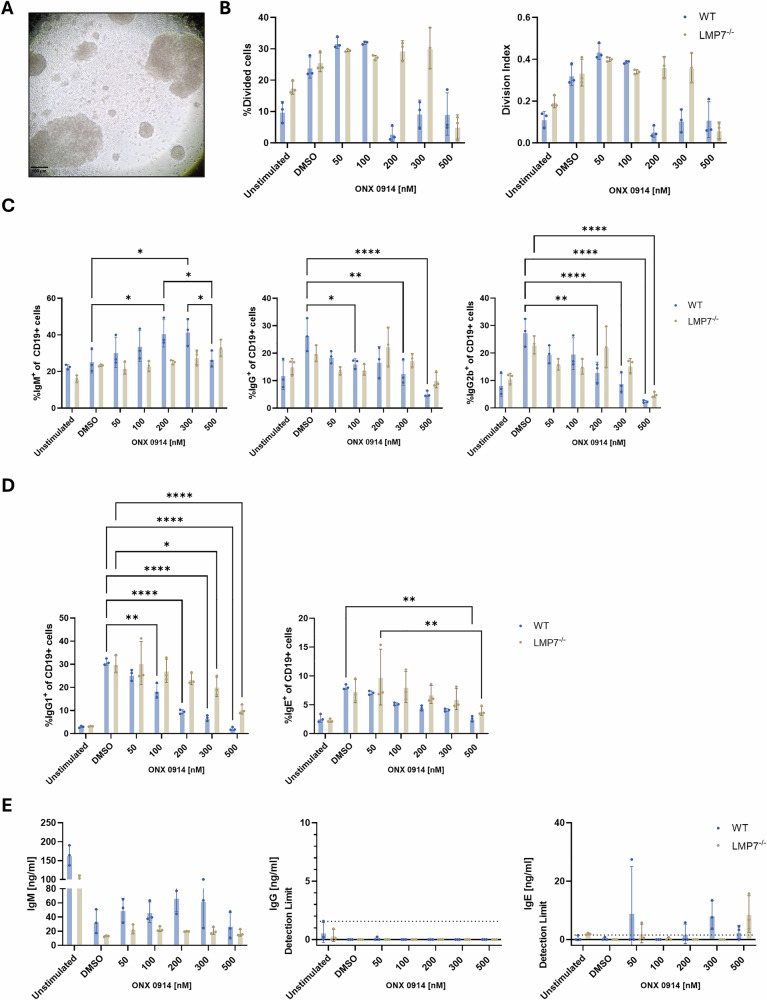
Immunoproteasome inhibition inhibits class-switch from IgM to IgG and IgE of primary mouse CD19^+^ cells in vitro. **A**–**E** Splenocytes from C57BL/6 or LMP7^-/-^ mice (*n* = 3) were magnetically enriched for CD19^+^ cells, stained with CFSE and stimulated by seeding on CD40L (1 µg/ml) coated 96-well plates with or without IL-4 (20 ng/ml). Cells were then treated with ONX 0914 and incubated for 72 h at 37 °C. Afterwards, cells were analyzed via flow cytometry for **B** proliferation and **C**, **D** expression of surface immunoglobulins. **A** Microscopic image of stimulated cells after 72 h (scale bar represents 100 µm). **B** Proliferation analysis. Percentage of divided cells (left panel) and division index (right panel) was assessed. Division index refers to the average number of cell divisions a cell of the original population has undergone. **C** Percentage of IgM, total IgG and IgG2b of CD19^+^ cells. **D** Percentage of IgG1 and IgE of CD19^+^ cells additionally treated with IL-4. **E** Secretion of antibodies into the supernatant was analyzed via ELISA. **B**–**E** Data from 3 biological replicates is presented as means ± SD. Statistics: two-way ANOVA *P < 0.05; **P < 0.01, ****P < 0.0001.

During the initial phase of an immune response, naïve B cells produce low-affinity IgM antibodies that provide only limited protection. Upon receiving antigenic stimulation and costimulatory signals from T cells, B cells proliferate and undergo class-switch recombination to produce other immunoglobulin isotypes, such as IgG and IgE. To investigate whether this process is affected by IP inhibition, we induced a class-switch in vitro using recombinant CD40 ligand (CD40L), a key molecule required for B cell activation and differentiation. Therefore, splenocytes from WT mice or LMP7^–/–^ mice were magnetically enriched for CD19^+^ cells, stained with carboxyfluorescein succinimidyl ester (CFSE) to monitor proliferation and cultured on CD40L-coated plates in the continuous presence of ONX 0914. Cells were incubated for 72 h to promote class-switch to IgG. Additionally, in a subset of samples, IL-4 was added to induce class switch to IgG1 and IgE. Afterwards, cells were analyzed by flow cytometry for surface expression of immunoglobulin isotypes, and culture supernatants were collected for ELISA analysis of secreted antibodies. Large clusters of B cells were observed after 72 h of culture, indicating a robust proliferation (Fig. 3A). Analysis of proliferation via CFSE dilution showed that treatment with ONX 0914 (≥200 nM) significantly reduced proliferation of WT cells compared to DMSO treated controls, indicated by reduced percentages of divided cells and a lower division index (Fig. 3B). In contrast, a similar reduction in proliferation in cells from LMP7^–/–^ mice was only seen at 500 nM ONX 0914, suggesting that the observed effects are IP inhibition specific (Fig. 3B). Surface staining for IgM revealed that treatment with ONX 0914 increased percentages of CD19^+^IgM^+^ WT cells, but not LMP7^–/–^ cells. On the other hand, proportion of surface IgG and IgG2b expressing CD19^+^ cells was significantly reduced by ONX 0914 treatment (Fig. 3C, representative flow cytometry data in Supplementary Fig. 4A). Co-stimulation with CD40L and IL-4 led to significantly increased percentages of IgG1^+^ and IgE^+^ CD19^+^ WT cells and LMP7^–/–^ cells. Continuous ONX 0914 treatment significantly reverted the percentage of IgG1^+^ WT cells (≥100 nm ONX 0914), while this effect was only seen at ≥300 nM in LMP7^–/–^ cells. For IgE^+^ cells, a significant reduction was only seen at 500 nM ONX 0914 in both WT and LMP7^–/–^ cells (Fig. 3D, representative flow cytometry data in Supplementary Fig. 4B). Analysis of secreted antibodies in the supernatant via ELISA revealed a reduction of IgM secretion in all CD40L-stimulated samples compared to unstimulated samples, likely due to the induction of isotype class-switching. ONX 0914 treatment partially reverted this reduction of IgM secretion in WT cells in an ONX 0914 concentration dependent manner. This effect of ONX 0914 treatment was not observed in the supernatant derived from LMP7^–/–^ cells (Fig. 3E). At 500 nM ONX 0914 treatment, IgM levels were reduced, suggesting induction of cell death instead of inhibition of class switch at higher concentrations of ONX 0914. Interestingly, very low levels of IgG and IgE were detected in the supernatant, indicating induction of isotype class-switch without switching from membrane-bound Ig to secreted Ig in the used setup. Together, the increased percentage of IgM^+^ cells and the enhanced IgM secretion in the presence of ONX 0914, along with the reduction in class-switched cells, strongly suggest that IP inhibition limits the ability of B cells to undergo effective class-switch recombination in vitro.

### In vivo IP inhibition does not affect antibody neutralization capacity

**Fig. 4:**
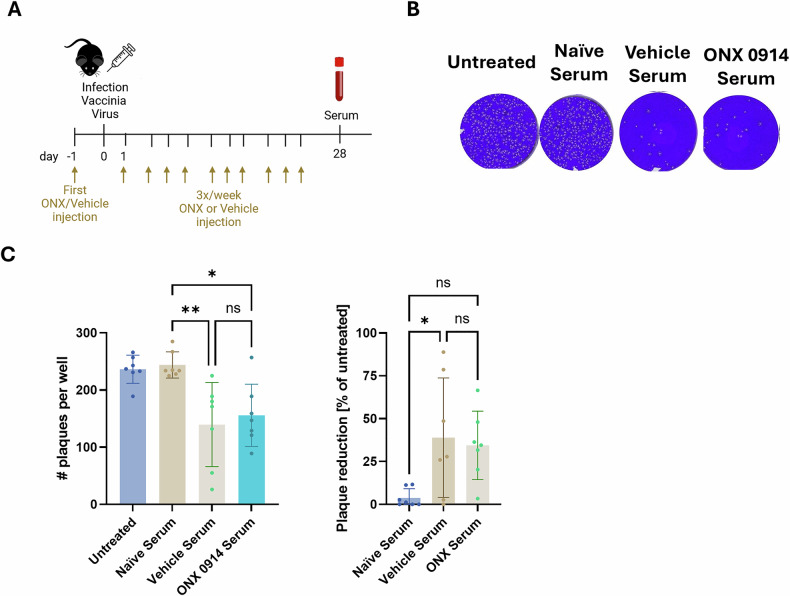
IP inhibition does not affect antibody neutralization capacity. In vivo **A**–**C** Female C57BL/6 mice (*n* = 7) were infected with 2 × 10^6^ pfu vaccinia WR i.p. Mice were treated three times per week with 10 mg/kg ONX 0914 s.c. starting one day prior to infection. Serum was collected on day 28 post infection. **A** Scheme of experimental setup. **B** Representative wells showing number of plaques in BSC-40 cells infected with VV-WR which was left untreated (w/o serum) or treated with serum from either naïve or infected mice (ONX 0914 or vehicle treated). **C** Quantification of plaques (left panel) and plaque reduction (right panel). Plaque reduction was calculated by setting the mean of plaques in untreated wells as 100%. Data derived from the serum of 7 different mice is presented as means ± SD. Statistics: one-way ANOVA. *P < 0.05; **P < 0.01.

In vitro treatment of B cells and antibody-secreting cells showed significant effects of IP inhibition on the UPR, induction of apoptosis and isotype class-switch. Therefore, we next investigated the effects of ONX 0914 treatment on antibody responses after infection in vivo. A single dose of 10 mg/kg ONX 0914 led to a complete inhibition of LMP7 in the spleen (Supplementary Fig. 5). To assess antibody responses after infection in vivo, female C57BL/6 mice were treated with 10 mg/kg ONX 0914 3 times a week starting one day prior to infection with vaccinia virus WR (VV-WR). On day 28 post infection (scheme shown in Fig. 4A), when protective antibody levels are typically established, mice were sacrificed for serum collection. Virus neutralization capacity of anti-vaccinia virus antibodies in the serum was determined in a plaque reduction neutralization assay. In this assay, serial dilutions of VV-WR were incubated with serum from VV-WR-infected mice (treated with either vehicle or ONX 0914), and the mixture was added to BSC-40 cells in vitro. As shown in the representative data in Fig. 4B, serum from mice previously infected with VV-WR reduced the number of plaque-forming units compared to untreated samples or samples treated with serum from naïve mice, indicating antibody-mediated virus neutralization. However, no significant effects of ONX 0914 treatment compared to vehicle-treated mice on virus neutralization capacity could be observed (Fig. 4C). These results indicate that in vivo, IP inhibition does not impair the development of functional antibody responses following VV-WR infection.

### Preventive treatment with ONX 0914 in an OVA vaccination model does not impact serum anti-OVA antibody titers

**Fig. 5:**
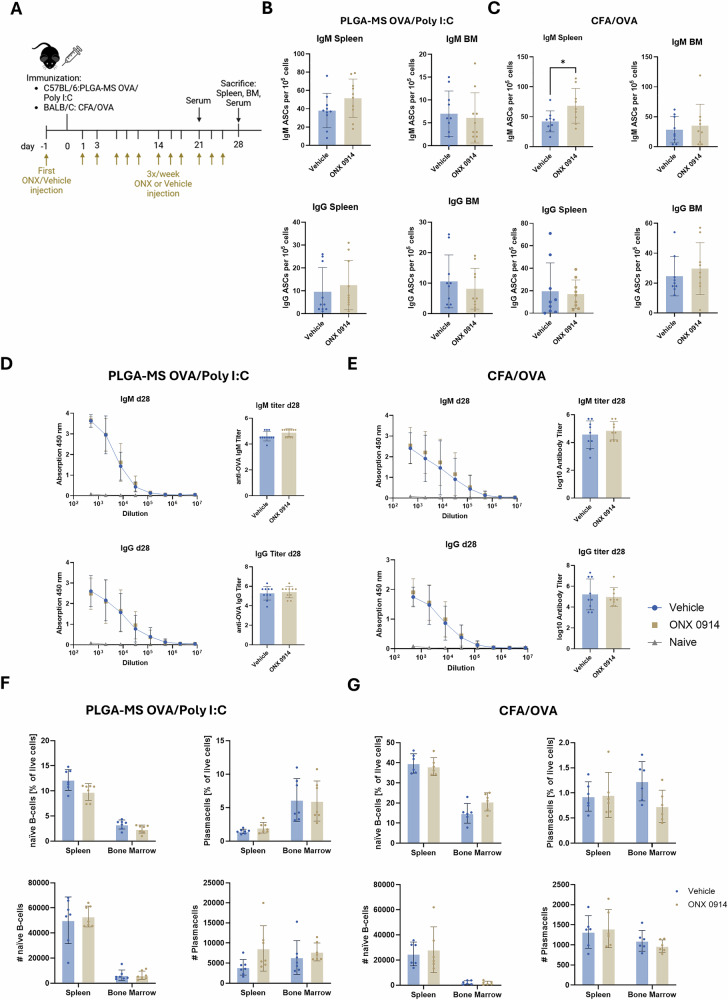
Preventive treatment with ONX 0914 in an OVA vaccination model does not impact serum anti-OVA antibody titers. C57BL/6 or BALB/c mice (*n* = 10) were s.c. immunized on day 0 with PLGA-MS OVA/poly I:C (**B**, **D**, **F**) or CFA/OVA (**C**, **E**, **G**), respectively. Mice were treated three times per week with 10 mg/kg ONX 0914 s.c. starting one day prior to immunization. **A** Scheme showing the experimental setup. **B**, **C** On day 28, bone marrow and spleen cells were isolated and ELISpot for anti-OVA IgM and IgG secreting cells was performed. Shown are the antibody-secreting cells per 10^5^ total cells. **D**, **E** Anti-OVA IgM and IgG titers in the serum derived from immunized (d28) or naïve mice were determined via ELISA. Antibody titers were defined as the decadic logarithm of the highest sample dilution exceeding the signal of the mean of naïve samples + 3x standard deviation (SD). **F**, **G** On d28 post immunization, bone marrow cells and spleen cells were analyzed via flow cytometry. Shown are the percentage and the absolute cell count of naïve B cells (CD19^+^IgM^+^B220^+^) and plasma cells (CD19^-^CD138^+^BLIMP1^+^). **B**–**G** Data is presented as means ± SD derived from 10 mice. Statistics: Student’s *t*-test *P < 0.05.

To further investigate the effect of IP inhibition on antibody responses in vivo, we employed two different vaccination models based on the model antigen ovalbumin (OVA). In the first model, C57BL/6 mice are immunized with PLGA-MS [42] containing OVA and the TLR-3 ligand Poly I:C. In the second model, BALB/c mice are immunized with OVA emulsified in Complete Freund’s Adjuvant (CFA). In both vaccination models, ONX 0914 treatment was initiated one day prior to immunization and continued 3 times per week until day 28 (Fig. 5A). First, we assessed the numbers of IgM or IgG antibody-secreting cells in spleen and bone marrow (BM) via ELISpot. Interestingly, IgM-secreting cells in the spleen of ONX 0914 treated mice were slightly increased compared to vehicle-treated mice, which reached statistical significance in CFA-OVA immunized mice. The amount of IgM-secreting cells in the BM, as well as IgG-secreting cells in spleen and BM was unaffected by ONX 0914 treatment in both immunization models (Fig. 5B, C). Next, antibody titers in the serum were analyzed on day 21 and day 28 via ELISA. No differences in both IgM and IgG titers between vehicle and ONX 0914 treated groups were observed either on day 21 (Supplementary Fig. 6) or on day 28 (Fig. 5D, E) post immunization. We additionally conducted flow cytometric analysis of B cells and plasma cells in the spleen and bone marrow of immunized mice to assess whether long-term treatment with ONX 0914 influenced those populations. However, we did not observe any significant changes in the total number or percentages of B cells (CD19^+^IgM^+^B220^+^) or plasma cells (CD19^-^CD138^+^BLIMP-1^+^), in the spleen or the bone marrow between ONX 0914- or vehicle-treated mice in both immunization models (Fig. 5F, G). Together, these results demonstrate that preventive ONX 0914 treatment does not adversely affect the development of a robust antibody response after immunization or alter the overall composition of B cell and plasma cell populations in vivo.

### Administration of ONX 0914 at later time points in OVA vaccination models does not impact serum anti-OVA antibody titers

**Fig. 6:**
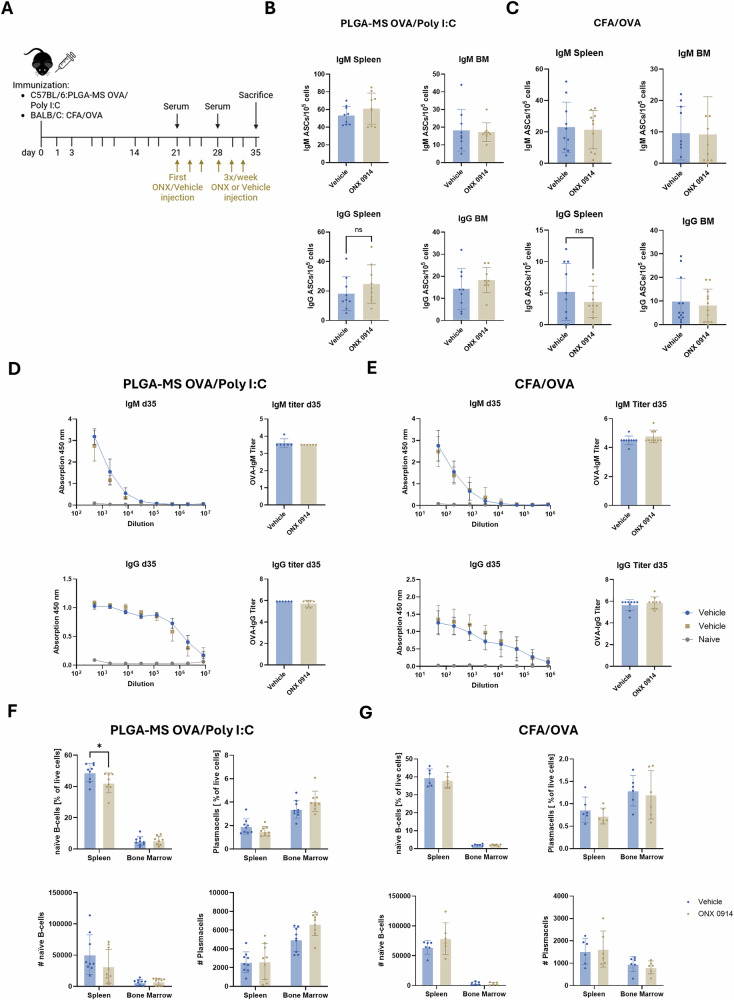
Administration of ONX 0914 at later stages in OVA vaccination models does not impact serum anti-OVA antibody titers. C57BL/6 or BALB/c mice (*n* = 10 per group) were immunized on day 0 with PLGA-MS OVA/poly I:C (**B**, **D**, **F**) or CFA/OVA (**C**, **E**, **G**), respectively. 21 days post immunization, mice were injected with 10 mg/kg ONX 0914 or vehicle 3x/week until sacrifice on day 35. Blood was drawn for serum antibody detection on day 21, day 28 and after sacrifice on day 35. **A** Scheme showing the experimental setup. **B**, **C** Bone marrow and spleen cells were isolated and ELISpot for anti-OVA IgM and IgG secreting cells was performed. **D**, **E** Serum from immunized or naïve mice was analyzed via ELISA and anti-OVA IgM and IgG titers were determined. Antibody titers were defined as the decadic logarithm of the highest sample dilution exceeding the signal of the mean of naïve samples + 3x standard deviation (SD). **F**, **G** Bone marrow and spleen cells were analyzed via flow cytometry for percentage and absolute count of naïve B cells (CD19^+^IgM^+^B220^+^) and plasma cells (CD19^-^, CD138^+^BLIMP1^+^). Data is presented as means ± SD. Statistics: Student’s *t*-test * **P < 0.01.

No major effect of IP inhibition in respect to antibody production could be observed in our two different OVA vaccination models. Next, we tested the effect of IP inhibition on antibody production in the two vaccination models in a “therapeutic” set-up. For this purpose, C57BL/6 mice or BALB/c mice were immunized with PLGA-MS poly I:C or CFA/OVA, respectively. Treatment with ONX 0914 was initiated on day 21 after immunization and continued 3 times per week until sacrifice on day 35 (Fig. 6A). ELISpot analysis of splenocytes and bone marrow cells showed no significant differences in the numbers of IgM- or IgG-secreting cells between vehicle- and ONX 0914-treated mice in both immunization models (Fig. 6B, C). Consistently, antibody titers in the serum analyzed via ELISA showed no differences in both IgM and IgG titers on day 35 post immunization between vehicle and ONX 0914 groups (Fig. 6D, E). Similar results were obtained when analyzing serum taken on day 21 and day 28 post immunization (Supplementary Fig. 7). Next, B cells and plasma cells in the spleen and bone marrow were analyzed by flow cytometry. Although the percentage of B cells (CD19⁺IgM⁺B220⁺) was slightly but significantly reduced in PLGA-MS poly I:C vaccinated mice, the B cells (CD19⁺IgM⁺B220⁺) and plasma cells (CD19⁻CD138⁺BLIMP-1⁺) mainly remained unaffected in the two different OVA vaccination models. (Fig. 6F, G). Together, these findings indicate that IP inhibition at later stages, when immunization-induced B cell responses are already established, does not negatively impact serum antibody titers and plasma cells.

## Discussion

IP inhibition has been shown to be effective in different antibody-mediated disease conditions in pre-clinical models [43, 44]. The LMP7/2-specific IP inhibitor ONX 0914 reduced antibody-mediated graft rejection in mouse heart and rat kidney transplantation models [44–46]. Thereby, IP inhibition has been shown to prevent transplant arteriosclerosis (TA) ensuing allograft rejection [37]. Apart from solid organ transplantation models, IP inhibition lowers autoantibody production by plasma cells in a mouse lupus erythematosus (SLE) model [36]. Moreover, zetomipzomib [21] is currently being investigated in a weekly dosing regimen in clinical trials for the treatment of autoimmune diseases, including dermatomyositis/polymyositis, systemic lupus erythematosus, lupus nephritis and autoimmune hepatitis [47]. In our study, we investigated the impact of selective IP inhibition using ONX 0914 on B cell function, antibody secretion, and class-switch recombination in vitro under continuous treatment. Furthermore, antibody responses were investigated after vaccination and viral infection in different in vivo models in a TIW dosing regimen.

In vitro, we first investigated the effect of IP inhibition on a newly generated LMP2/LMP7 inducible human lymphoma B cell line. After induction with 4-OHT, standard proteasomes are replaced by IPs in these cells. Our observation of increased CHOP and spliced XBP1 mRNA expression, together with the accumulation of poly-ubiquitinated proteins, indicates an ONX 0914 induced activation of UPR. The UPR is an adaptive response to ER-stress, which is induced after accumulation of unfolded or misfolded proteins in the ER lumen, aiming to reduce protein accumulation to restore normal cell function. However, if protein aggregation is not resolved, UPR signaling can finally lead to apoptotic cell death [48, 49]. Due to high levels of antibody synthesis, ASCs are highly dependent on functional proteasomal degradation to maintain protein homeostasis [2, 27, 29]. Therefore, it was not surprising to see induction of apoptosis after UPR activation in IP expressing LCL721-G27 cells, which constantly produce significant levels of IgG. Additionally, IP inhibition similarly led to apoptosis of CD19-positive cells in LPS-activated primary murine splenocytes and human PBMCs. Of note, whereas previous studies used a 1-h pulse treatment to prevent unspecific binding to standard proteasome subunits [13, 21], we used continuous IP inhibitor treatment in our study. However, using cells derived from LMP7^–/–^ mice, we could confirm that the effects of ONX 0914 were IP selective. Interestingly, continuous treatment of splenocytes with the immunoproteasome inhibitor LU-005i for 3 days in the absence of LPS did not induce cell death in CD19^+^ cells [50], indicating that activation is required to affect B cell differentiation. Induction of apoptosis of CD19^+^ cells in LPS-activated primary murine splenocytes and hPBMCs is consistent with prior studies showing that proteasome inhibition with broad-spectrum proteasome inhibitors can induce apoptosis in ASCs, as has been shown in multiple myeloma but also normal human plasma cells [28, 31, 51, 52].

Our data indicate that ONX 0914 interferes with the process of immunoglobulin class-switch recombination. When we induced class switch of CD19^+^ primary splenocytes via CD40L stimulation, ONX 0914 treatment resulted in an increased percentage of IgM-positive cells and a concurrent decrease in IgG- and IgE-positive cells. Furthermore, IgM secretion was enhanced with increasing ONX 0914 concentrations, indicating that class switch to IgG or IgE was suppressed. This finding is consistent with another study by Muchamuel et al., which has shown that differentiation of human B cells into plasmablasts was impaired by IP inhibition [21]. Interestingly, at 300 nM and 500 nM ONX 0914 IgM secretion was not enhanced anymore, suggesting apoptosis was induced at these concentrations as observed before. How ONX 0914 inhibits the molecular events that lead to class-switching was not further clarified in this study; however, class switch is highly dependent on the expression of activation-induced deaminase (AID) [53, 54]. AID has been shown to be a key regulator of class-switch recombination, as evidenced by mutations in AID leading to hyper-IgM syndrome, which is characterized by an increase in serum IgM and lack of class switched immunoglobulins [55, 56]. Interestingly, expression of AID has previously been shown to be regulated by nuclear factor (NF)-*κ*B [57, 58]. Activation of NF-*κ*B, in turn is dependent on proteasomal degradation of its inhibitory subunit IκB, and proteasomal inhibition has thereby been shown to prevent activation of NF-*κ*B [59–61]. Given that, the inhibition of NF-*κ*B activation could result in reduced AID expression, providing a potential mechanism for inhibition of class-switch recombination via proteasome inhibition. However, ONX 0914 did not inhibit NF-*κ*B activity in a reporter cell line at selective concentrations [13] and IP subunit deficiency had no influence on the canonical pathway of NF-κB activation [62]. Notably, previous studies showed that LMP7 subunit deficiency alone can already have suppressive effects on inflammatory processes, such as the differentiation of helper T cells into Th1 and Th17 cells [10]. However, our results indicate that LMP7-deficient cells can undergo class-switch in vitro in a similar manner to WT cells, suggesting that inhibition but not absence of LMP7 impairs class-switch recombination. It has been shown that activation of T and B cells is suppressed in the presence of IP inhibition [12]. It seems obvious that such a mechanism can contribute to the observed inhibition in class switching but this remains to be investigated.

In contrast to our in vitro data, effects of IP inhibition in vivo in vaccina virus-infected mice and in two different OVA-based vaccination models was minor in respect to B cells and related functions. Although we confirmed effective IP inhibition in vivo, as demonstrated by a shift in molecular size of LMP7 in splenic cells, the antibody neutralization capacity to vaccinia virus after infection was not affected by ONX 0914 treatment. In our OVA vaccination model, when ONX 0914 was applied from the beginning, we did observe a significant increase in anti-OVA IgM-secreting cells in the spleen of ONX 0914 treated mice, suggesting that B cells might be impaired in their ability to undergo class-switch recombination toward higher-affinity IgG isotypes in vivo. This observation aligns with our in vitro findings on class switching. However, despite the increased numbers of IgM-secreting cells, we did not detect corresponding changes in serum antibody titers. Neither an increase in IgM nor a decrease in IgG serum titers was apparent in all applied in vivo settings. This discrepancy indicates that modest alterations in the number of ASCs may not be sufficient to affect systemic antibody levels, particularly given the massive antibody output from plasma cells following immunization or infection. One potential explanation for the difference to the in vitro findings is the differential activation state of cells in vitro and in vivo. When we treated primary murine splenocytes with LPS, massive production of IgM and IgG antibodies was observed compared to untreated cells, which might produce a level of protein stress that is not reached under in vivo conditions. Additionally, in vivo, there could be compensatory mechanisms that rescue cells from IP inhibition mediated apoptosis. B-cell and plasma cell responses in vivo are regulated not only by cell-intrinsic factors but also by signals from T cells and the surrounding microenvironment. In vivo, several factors such as APRIL, IL-6 and BAFF, which are expressed by macrophages, monocytes, dendritic cells or bone marrow stromal cells, act as pro-survival signaling molecules on B cells and plasma cells [63, 64]. These factors are only present in whole organisms and could mitigate the direct pro-apoptotic effects of IP inhibition that were observed in isolated B cells in vitro. Another explanation could be that continuous treatment with ONX 0914 in vitro might not reflect in vivo persistence of ONX 0914. This is supported by another study, which showed that in vitro differentiation of human CD19^+^ peripheral B cells to plasmablasts was impaired after pulse-treatment with ONX 0914, but did not lead to apoptosis [21]. Pulse-treatment instead of continuous treatment might more accurately reflect the half-life of ONX 0914 in vivo, which might contribute to the different outcomes between in vitro and in vivo experiments in this study.

Our in vivo findings on antibody production deviate from previous studies on autoimmunity and organ transplantation [43, 65] which showed reduced production of auto- and allo-antibodies. Ichikawa et al. found beneficial effects of IP inhibition in lupus-prone mice due to reduced type I IFN activation and autoantibody production by plasma cells [36]. Interestingly, they observed greater reductions in autoreactive compared to total IgG ASCs, indicating that autoreactive ASCs might be more susceptible to IP inhibition due to higher rate of antibody production [36]. Similar results in SLE were found with the clinical candidate IP inhibitor KZR-616 [21]. In a model of Graves hyperthyroidism, another autoimmune disease mediated by production of autoantibodies [66], treatment of mice with 20 mg/kg ONX 0914 showed no effect on anti-TSHR antibody titers [67]. These observations indicate that IP inhibition may act in a context-dependent manner on B cells and ASCs, with only highly-secretory ASCs being susceptible to IP inhibition. This is in line with our in vitro data, where splenocytes activated with LPS were highly susceptible to continuous ONX 0914 treatment. Similar to auto-antibodies, IP inhibition reduced allo-antibodies in a chronic antibody-mediated kidney transplantation model in rats [44, 46] and in a rat aorta transplantation model [37]. Interestingly, inhibition of one IP subunit was not enough to decrease IgG-secreting cells in the spleen and bone marrow, but inhibition of LMP7 and LMP2 was required, indicating that a certain degree of proteasome inhibition must be achieved to affect antibody-secreting cells [37]. Our study is in agreement with a previous study using KZR-616 [21]. In this study, weekly KZR-616 treatment did not alter the KLH antibody titers after vaccination with keyhole limpet hemocyanin in mice and monkeys [21].

Overall, our in vivo data support the notion that IP inhibitors might have a therapeutic window in immune-mediated diseases, allowing for the attenuation of inflammation without severely compromising protective antibody responses. Nevertheless, there are certain limitations to our study demanding further investigation. In healthy volunteers, subcutaneous administration of the clinical candidate KZR-616 showed rapid absorption with a *T*_max_ < 1 h and rapid clearance with a *t*_1/2_ < 4 h [68]. In contrast, in our in vitro setup cells were continuously treated with ONX 0914, allowing inhibition of newly built immunoproteasomes. In vivo in mice, we used a TIW dosing regimen. Hence, new immunoproteasomes built in the time between different administrations are fully functional, partially explaining differential sensitivity between in vitro and in vivo settings. In summary, our findings suggest that while the IP plays a critical role in maintaining protein homeostasis and regulating B cell function in vitro, its inhibition does not impair the generation of functional antibody responses in vivo after viral infection and vaccination. These results indicate that IP inhibitors can be safely used to treat autoimmune diseases without harming humoral immunity after vaccination and viral infection.

## Materials and methods

### Animals

C57BL/6J and BALB/c mice were originally purchased from Charles River (Sulzfeld, Germany) and further bred in the animal facility of the University of Konstanz. LMP7^–/–^ mice [69] were kindly provided by Dr. John J. Monaco (Department of Molecular Genetics, Cincinnati Medical Center, Cincinnati, OH, USA). The animals were kept under specific pathogen-free conditions in air-conditioned rooms with a controlled temperature of 22 °C and 55% relative humidity on a 12 h light/dark cycle. Animals had ad libitum access to standard, autoclaved laboratory animal diet and tap water. Male and female mice were used at 6–14 weeks of age. At the beginning of the experiment, mice were randomly assigned to treatment groups. To avoid sex bias, groups were stratified to contain equal numbers of male and female animals. No blinding was performed. Investigators were aware of group allocation during the experiments and outcome assessment. Animal experiments have been conducted in compliance with ethical standards of German and EU guidelines after approval by the animal experimentation ethics committee of the Review Board of the Governmental Presidium Freiburg, Germany with the approval numbers T-21/03TFA, T-24/02TFA, G-23/059 or the Ethics Committee of Chongqing University Cancer Hospital (CZLS2020289-A).

### Cell lines and culture media

The LMP2/LMP7 inducible cell line LCL721-G27 was generated as follows. A tamoxifen-based system composed of pFU and pGev16 (kind gift of Thomas Brunner, University of Konstanz) [70] was used for inducible expression of LMP2 and LMP7. LCL721.174 cells [50], which lost the genomic region encoding the genes for LMP2 and LMP7, were first transduced by addition of the lentiviral particles of pGev16. Next, LCL721.174-Gev16 cells were simultaneously transduced with pFU-LMP2 and pFU-LMP7 and limiting dilution was performed to obtain single-cell clones which were tested for the expression of both subunits by immunoblotting (see below). The resulting cell lines were termed LCL721-G27. 7 days post tamoxifen treatment, these cells only express immunoproteasomes in contrast to untreated cells expressing standard proteasomes (Supplementary Fig. 1). Purification of proteasomes and 2D-gel electrophoresis used for Supplementary Fig. 1 was performed as previously described [71, 72]. The LMP2/LMP7 inducible cell line LCL721-G27 was cultured in Iscove’s modified Dulbecco medium (IMDM) supplemented with 10% FCS and 1% P/S. 4-hydroxy-tamoxifen (4-OHT; Sigma Aldrich) was dissolved in ethanol and used at 20 nM to induce expression of IP. The BSC-40 cell line was cultured in Minimum Essential Medium (MEM) containing 5% FCS and 1% P/S. Cell culture reagents were purchased from ThermoFisher. All cells were cultured in a humid chamber at 37 °C and 5% CO_2_. All cell lines used were tested negative for mycoplasma contamination.

### In vitro proteasome inhibition

LCL721-G27 cells were induced for at least 1 week with 4-OHT before any experiments were performed. For determining accumulation of poly-ubiquitin conjugates, cells were continuously treated for 6 h with increasing concentrations of ONX 0914 (Kezar Life Sciences), before being lysed for immunoblotting. For determining cell death and antibody secretion, cells were continuously treated for 24 h with increasing concentrations of ONX 0914 (Kezar Life Sciences) or DMSO as solvent control. Cell supernatant was stored at –80 °C until further analysis. Cells were stained with APC Annexin V and PI-staining solution (BioLegend) for flow cytometric analysis.

### Analysis of unfolded protein response via quantitative RT-PCR

LCL721-G27 cells were continuously treated for 16 h with increasing concentrations of ONX 0914, and cells were pelleted before continuing with RNA-extraction. RNA was isolated from the cell pellets using the RNeasy Mini kit (Qiagen). After purity assessment with a NanoVue (GE Healthcare), cDNA was synthesized using Biozym cDNA synthesis kit (#331470L). Real-time RT-PCR (Biozym Blue S′Green Kit) was performed in a Biometra TProfessional Thermocycler (Analytik Jena). Primers used are listed in Supplementary Table 1.

### Immunoblotting

LCL721-G27 cells were lysed in RIPA buffer (50 mM Tris-buffered HCl, pH 7.5 containing 150 mM NaCl, 1% NP-40, 0.5% SDS) for 20 min on ice and protein content was determined by BCA assay (ThermoFisher). 20 µg protein lysate was separated by SDS-PAGE and transferred onto a nitrocellulose membrane. Expression of IP subunits was analyzed by incubation with antibodies directed against LMP7, LMP2 and MECL-1 as well as the standard subunits β1c, β2c and β5c (Supplementary Table 2). Anti-γ-tubulin and anti-β-actin served as loading controls. Accumulation of poly-ubiquitin conjugates was assessed via incubation with an anti-ubiquitin antibody. IRDye800CW goat anti-rabbit or anti-mouse and IRDye680RD goat anti-mouse or anti-rabbit (LI-COR) were used as secondary antibodies. Signals were analyzed with the LI-COR Odyssey Imager and the Image Studio Lite Version 5.2.

### Organ collection and isolation of single-cell suspension

Mouse spleens were dissected, and a single cell suspension was prepared by mechanical disruption. Cells were resuspended in IMDM medium supplemented with 10% FCS, 1% P/S and 50 µM β-mercaptoethanol. Mouse femur and tibia were acquired and disinfected for 5 min in 70% ethanol. Bone marrow was obtained via flushing with a 25G 5/8 syringe and resuspended in RPMI medium supplemented with 10% FCS, 1% P/S and 50 µM β-mercaptoethanol. For ELISpot analysis splenocytes or bone marrow cells were treated with NH_4_Cl (1.66% w/v in H_2_O) to remove erythrocytes.

### Serum collection

Blood was collected from sacrificed mice by cardiac puncture and transferred into serum collection tubes (Sarstedt). Samples were centrifuged at 10,000 × *g* for 5 min to separate the serum, which was frozen at –80 °C till further analysis. Antibody concentrations were measured via ELISA.

### In vitro activation of splenocytes

Splenocytes were depleted of erythrocytes and seeded at 5 × 10⁵ cells per well in 96-well plates. Cells were cultured in the presence of 10 µg/ml LPS (*Escherichia coli* 0111:B4, Sigma) and increasing concentrations of ONX 0914 (Kezar Life Sciences) or DMSO as a solvent control. After 72 h of incubation at 37 °C, supernatants were collected for ELISA analysis of total IgM and IgG. Cells were subsequently harvested for flow cytometric analysis.

### In vitro class switch of primary mouse splenocytes

Splenocytes from C57BL/6 mice were enriched for CD19⁺ cells using CD19 MicroBeads (Miltenyi Biotec) according to the manufacturer´s protocol and stained with 5 µM CFSE (BioLegend) prior to seeding. For IgG class switching, 4 × 10^4^ cells per well were plated in 96-well plates pre-coated overnight with 1 µg/ml (in PBS) recombinant CD40L (BioLegend). For IgE class switching, cells were additionally stimulated with 20 ng/ml IL-4. Following treatment with increasing concentrations of ONX 0914 (Kezar Life Sciences) for 72 h at 37 °C, supernatants were collected for ELISA, and cells were harvested for flow cytometric analysis of surface immunoglobulin expression. Proliferation was assessed using FlowJo’s proliferation analysis tools (FlowJo™ v10.9 (BD Biosciences)).

### In vivo proteasome inhibition

ONX 0914 (10) (Kezar Life Sciences) was formulated in a solution of 10% sulfobutylether-β-cyclodextrin and 10 mM sodium citrate (pH 6; vehicle). 10 mg/kg was administered subcutaneously in the neck 3x per week. Control mice received the same volume of vehicle.

### Vaccinia virus infection

C57BL/6 mice were infected intraperitoneally with 2 × 10⁶ plaque-forming units (pfu) of Vaccinia Virus WR (VV-WR). ONX 0914 or vehicle was administered at the indicated time points. Serum was collected 28 days post infection, heat-inactivated at 56 °C for 30 min to inactivate complement, and stored at −80 °C for subsequent neutralization assays.

### Plaque reduction neutralization assay

For determination of neutralization capacity of serum from VV-infected mice, a plaque assay using BSC-40 cells was employed. Therefore, 1.5 × 10^5^ BSC-40 cells were seeded onto 24-well plates the day before adding VV-WR of known concentration. Tenfold dilutions of VV-WR were prepared and added to the BSC-40 cells along with diluted serum (1:100) of VV-WR-infected mice who received either ONX 0914 or vehicle, or serum from naïve mice. After 1 h of incubation, 1 mL MEM was added, and the cells were incubated for an additional 24 h. After removing the medium, plaques were stained with 0.5% (w/v) crystal violet for 15 min, washed, and counted. The neutralization capacity was calculated as the percentage reduction in plaque numbers relative to wells incubated without serum.

### Vaccination

For vaccination studies, mice were immunized either with poly(lactic-co-glycolic acid) (PLGA) microspheres (MS) containing OVA/Poly I:C or with Complete Freund’s Adjuvant (CFA)/OVA. In the PLGA MS model, mice received 5 mg of PLGA MS OVA/poly I:C in PBS subcutaneously at the base of the tail. In the CFA/OVA model, mice were injected subcutaneously at the flank with 100 µl of CFA containing 250 µg OVA per mouse.

### Enzyme-linked immunosorbent assay (ELISA)

#### OVA-specific antibody titers in serum

For determination of OVA-specific antibody titers in serum, high-binding microplates (Greiner) were coated overnight at 4 °C with 0.1 mg/ml OVA protein (Merck) in PBS. After washing, serial dilutions of mouse sera were added and incubated at room temperature for 2 h. Plates were then incubated with biotinylated goat anti-mouse IgM or IgG antibodies (MabTech) for 1 h, followed by a 30 min incubation with streptavidin-HRP (BD Biosciences). The reaction was developed with TMB substrate (ThermoFisher), and absorbance was read at 450 nm using an Infinite M200PRO plate reader (TECAN). Antibody titers were defined as the highest dilution that exceeded the mean signal of naïve samples plus three standard deviations.

#### Total IgM, IgG and IgE antibodies from cell supernatants

Total IgM, IgG, and IgE concentrations in cell culture supernatants were measured using the Mouse IgM Uncoated ELISA Kit, Mouse IgG Uncoated ELISA Kit, and Mouse IgE Uncoated ELISA Kit (Invitrogen), respectively, following the manufacturer’s instructions.

### ELISpot

To analyze IgM and IgG production by OVA-specific plasma cells, ELISpot assays were performed using MultiScreen^®^HTS PVDF Filter Plates (Merck Millipore) pre-coated overnight at 4 °C with 0.1 mg/ml OVA protein (Merck) in PBS. Freshly isolated splenocytes or bone marrow cells, after erythrocyte depletion, were seeded at 5 × 10⁵ cells per well and incubated at 37 °C in a humidified 5% CO_2_ atmosphere for 18 h. After washing, the plates were incubated with biotinylated secondary antibodies (MabTech) specific for IgM or IgG for 2 h, followed by a streptavidin–ALP conjugate (BD Biosciences) for 30 min. The plates were then developed with BCIP^®^/NBT substrate (Merck) until distinct spots were visible. Plates were dried overnight, and spots were counted using the ImmunoSpot^®^ S6ULTRA analyzer and ImmunoSpot^®^ software version 7.0.33.1 (CTL Europe).

### Flow cytometry of primary mouse cells

Cells were first depleted of erythrocytes and then stained for viability using the Zombie UV™ Fixable Viability Kit (BioLegend) in PBS, following the manufacturer’s instructions. After one wash, cells were incubated with TruStain FcX™ (anti-mouse CD16/32; BioLegend) for Fc receptor blocking. Subsequently, cells were stained with different antibody cocktails (see Supplementary Table 1). For antibodies conjugated to biotin, cells were further incubated with streptavidin PE-Cy7 (1:1000; BioLegend). For intracellular staining, cells were fixed in 4% paraformaldehyde, permeabilized with a PBS-based buffer containing 0.1% saponin, 2% FCS, 2 mM EDTA, and 2 mM NaN_3_, and stained with anti-BLIMP-1 antibodies. Data were acquired on a CytoFLEX LX (Beckman Coulter) and analyzed using FlowJo™ v10.9 (BD Biosciences).

### Statistics

Data was analyzed using GraphPad Prism version 10 and depicted as mean ± standard deviation (SD). Individual data points represent different mice or independent in vitro experiments. Normality tests (Kolmogorov–Smirnov and Shapiro–Wilk) were performed to check for Gaussian distribution. In case of normal distribution, Student’s *t*-test, one- or two-way-ANOVA, or multiple *t*-test was performed to determine statistical significance of differences. Non-normally distributed data was analyzed by Kruskal–Wallis test or Mann–Whitney test. Statistical significance was achieved when *p* < 0.05; *p < 0.05, **p < 0.01, ***p < 0.001, and ****p < 0.0001. Sample sizes were chosen based on prior studies using similar assays and experimental systems (e.g. [73, 74]). For animal experiments, sample size was determined based on the primary endpoint of anti-OVA antibody titers between groups, using ANOVA to ensure adequate power to detect differences.

## Supplementary information

Supplementary Information

## Data Availability

All data reported in this paper will be shared by the corresponding author upon request. This paper does not report new code. Any additional information required to reanalyze the data reported in this paper is available from the corresponding author upon request.
