# Supplementary material for: Immunoproteasome inhibition triggers protein stress and apoptosis in cells of B cell lineage without impairing vaccination-induced antibody responses
Source: Cell Death Discov. 2025 Nov 24;11:545. doi: 10.1038/s41420-025-02818-w (PMC12644917; doi:10.1038/s41420-025-02818-w)
Supplement: Supplementary file 1 — Supplementary Information [file 41420_2025_2818_MOESM1_ESM.pdf]

Supplementary Information

Immunoproteasome inhibition triggers protein stress and apoptosis in cells of B-cell lineage without impairing vaccination-induced antibody responses

Dennis Mink[1,2], Franziska Oliveri[2], Julia Otto[2], Nazlim Kutsi[2], Carolina Gonzalez Siebold[2], Tony Muchamuel[3], Jun Li[4], Michael Basler[1,2*]

[1] Institute of Cell Biology and Immunology Thurgau (BITG) at the University of Konstanz, CH-8280 Kreuzlingen, Switzerland

[2] Division of Immunology, Department of Biology, University of Konstanz, D-78457 Konstanz, Germany

[3] Department of Research, Kezar Life Sciences, South San Francisco, California, USA

[4] Department of Urological Oncology Surgery, Chongqing University Cancer Hospital, 400030 Chongqing, China

* Corresponding author: Michael Basler

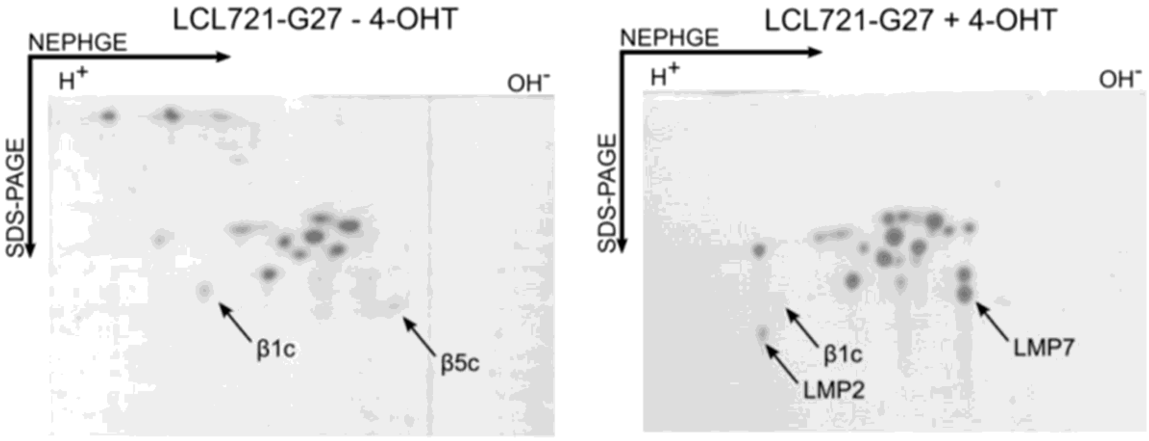

Supplementary Fig. 1: 4-OHT induced immunoproteasome expression in the newly generated LCL721-G27 cell line. 20S proteasome was purified from LCL721-G27 cells cultured for 8 days with (right side) or without (left side) 4-OHT and analyzed by 2-dimensional gel electrophoresis (SDS-PAGE and nonequilibrium pH gel electrophoresis – NEPHGE). Arrows indicate respective subunits.

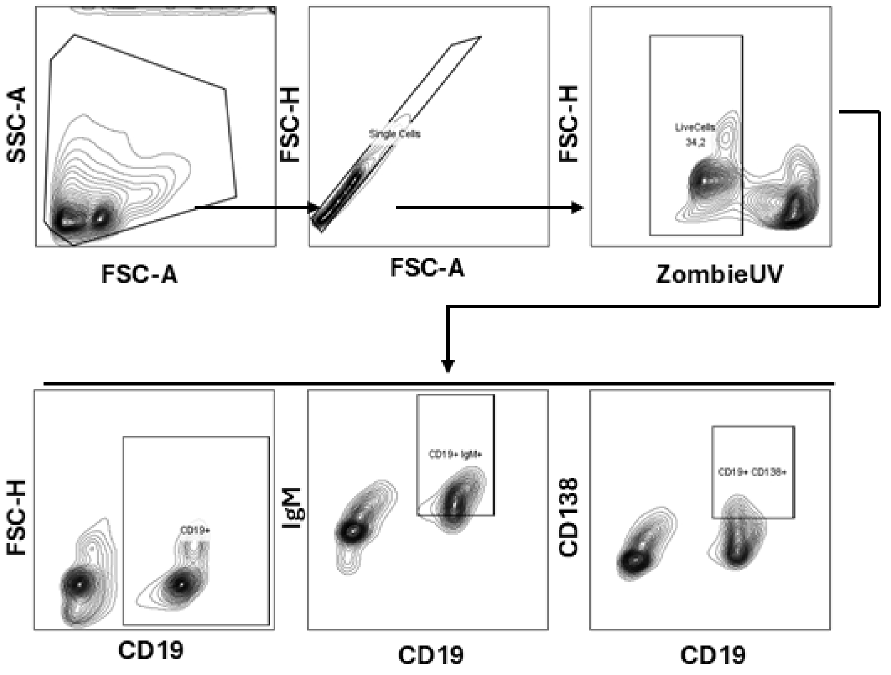

Supplementary Fig. 2: Gating strategy for B-cell populations after stimulation of mouse splenocytes with LPS. Mouse splenocytes were activated with 10 µg/ml LPS and treated with different concentrations of ONX 0914. After 72 h, cells were analyzed via flow cytometry. After the exclusion of debris, doublets and dead cells (ZombieUV), cells were gated for $CD19^+$ cells (left panel), $CD19^+IgM^+$ cells (middle panel) or $CD19^+CD138^+$ cells (right panel).

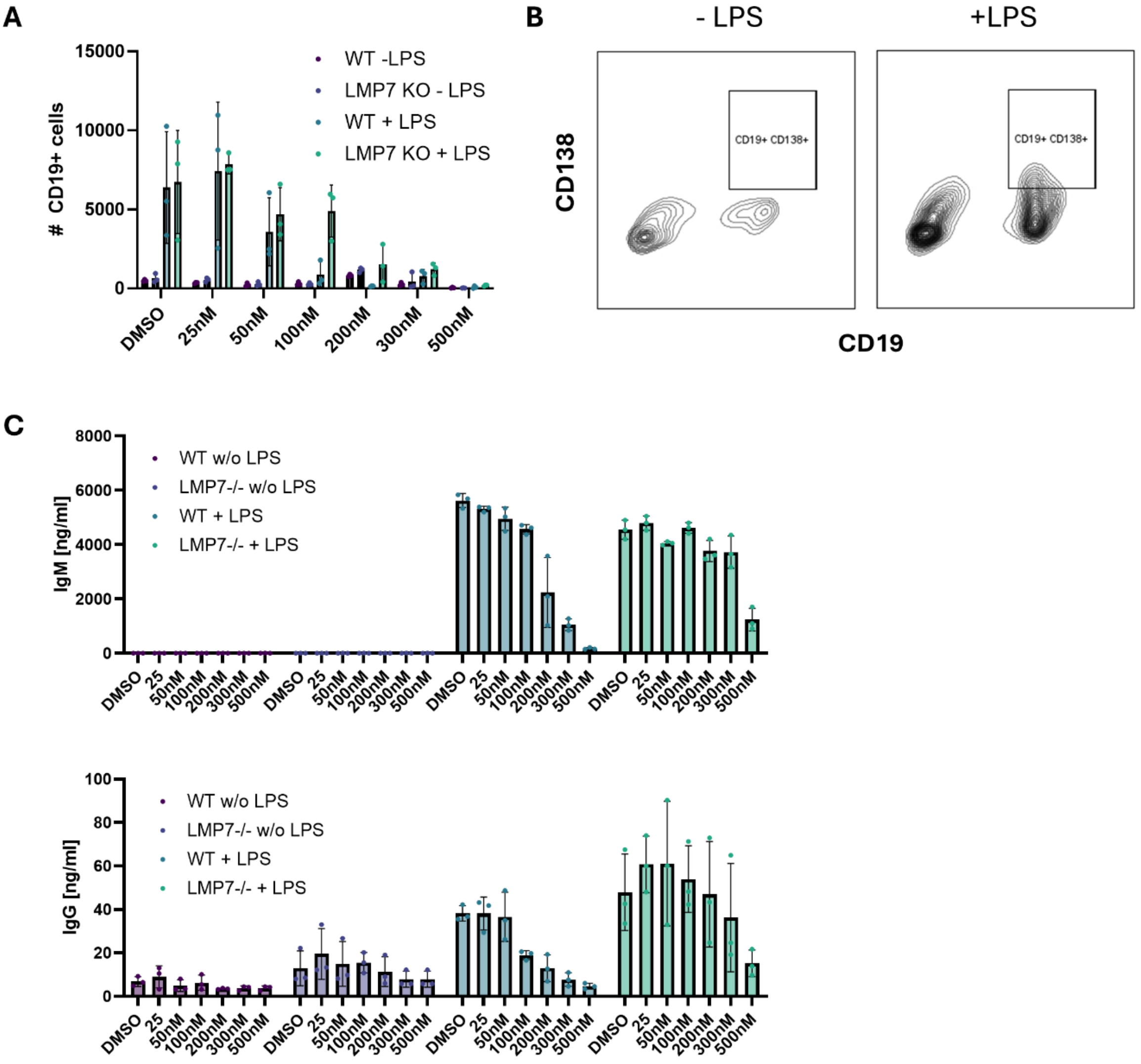

Supplementary Fig. 3: Analysis of B-cell populations and antibody secretion from splenocytes after 72 h of ONX 0914 treatment with or without LPS activation. Mouse splenocytes derived from wild type (WT) mice or LMP7-deficient mice (LMP7$^{-/-}$) were treated with or without 10 µg/ml LPS and indicated concentrations of ONX 0914. After 72 h, cells were analyzed via flow cytometry. **(A)** Absolute count of CD19^{+} cells, comparing LPS-treated to untreated cells (-LPS). **(B)** Exemplary data from flow cytometry comparing the CD19^{+}CD138^{+} population between LPS treated and untreated cells. **(C)** IgM (top panel) and IgG (lower panel) antibodies quantified in the cell supernatant via ELISA.

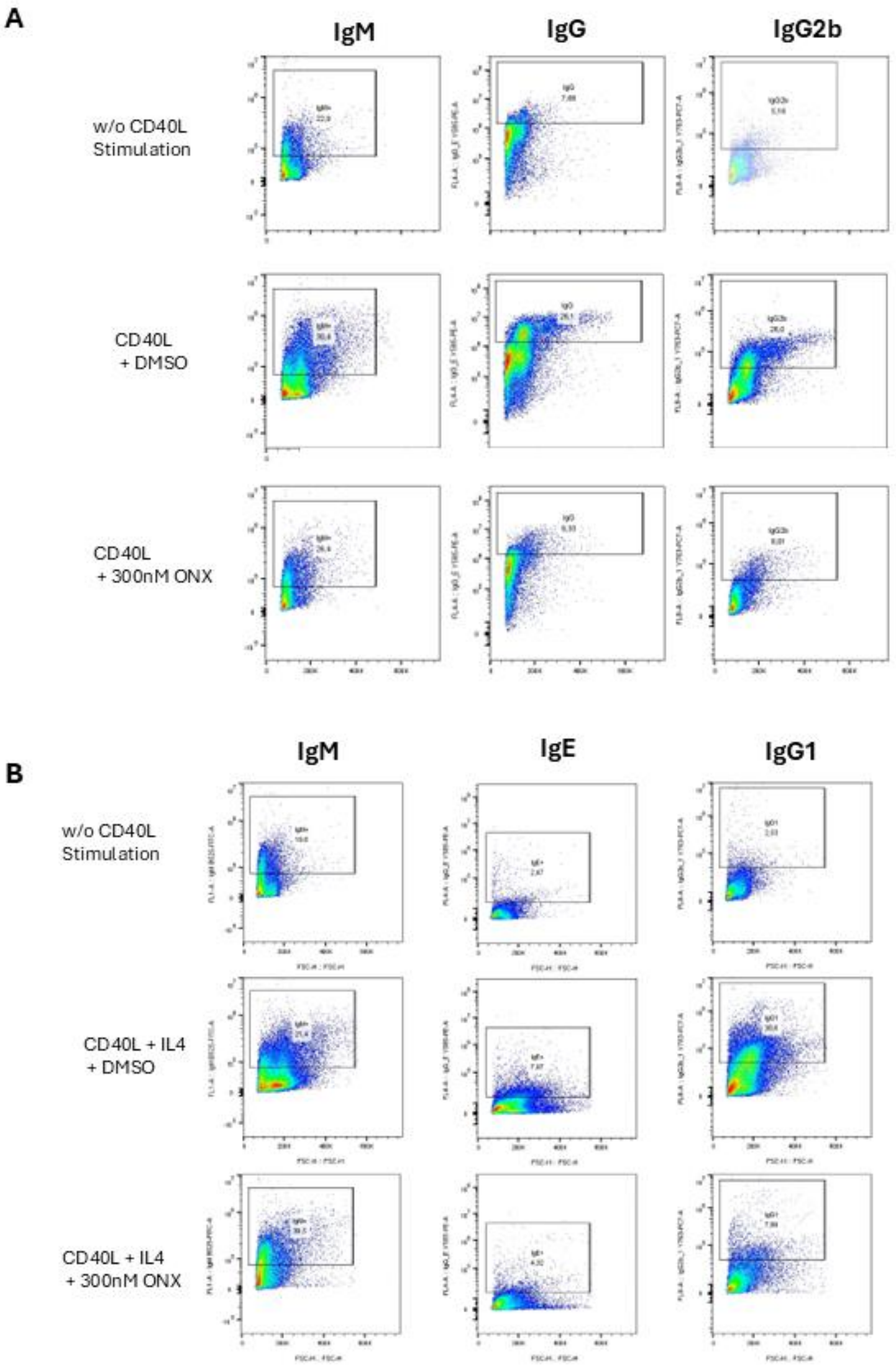

Supplementary Fig. 4: IP-inhibition reduces class-switched cells after induction via CD40L. C57BL/6 mouse splenocytes were magnetically enriched for $CD19^+$ cells, stained with CFSE and stimulated by seeding on CD40L coated plates with or without (w/o) IL-4 (20 ng/ml). Cells were treated with ONX 0914 or DMSO and incubated for 72 h. Afterwards, cells were analyzed via flow cytometry. **(A)** Representative plots showing percentage of IgM^+, IgG^+ and $IgG2b^+$ cells of $CD19^+$ cells at indicated conditions (left) **(B)** Representative plots showing percentage of IgM^+, IgE^+ or $IgG1^+$ cells of $CD19^+$ cells at indicated conditions (left)

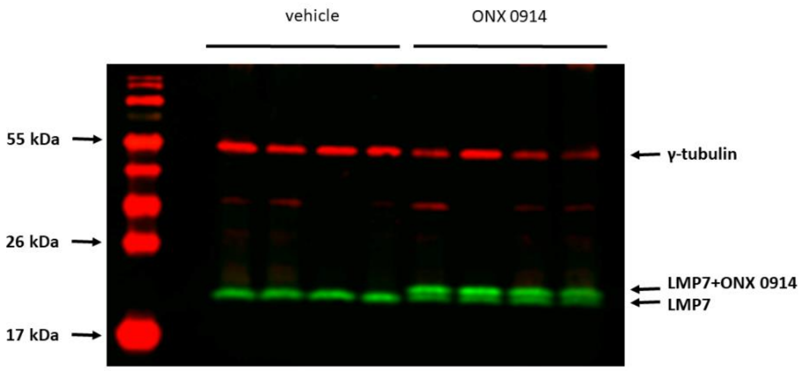

Supplementary Fig. 5: Confirmation of IP-inhibition in splenocytes after in vivo treatment of mice with ONX 0914. Mice were injected with 10 mg/kg ONX 0914 (n=4) or vehicle (n=4) one day before being sacrificed. Splenocytes were isolated and lysed for immunoblotting against LMP7. An increase of the molecular weight of LMP7 in ONX 0914 treated mice confirms successful binding of ONX 0914 to LMP7 *in vivo.*

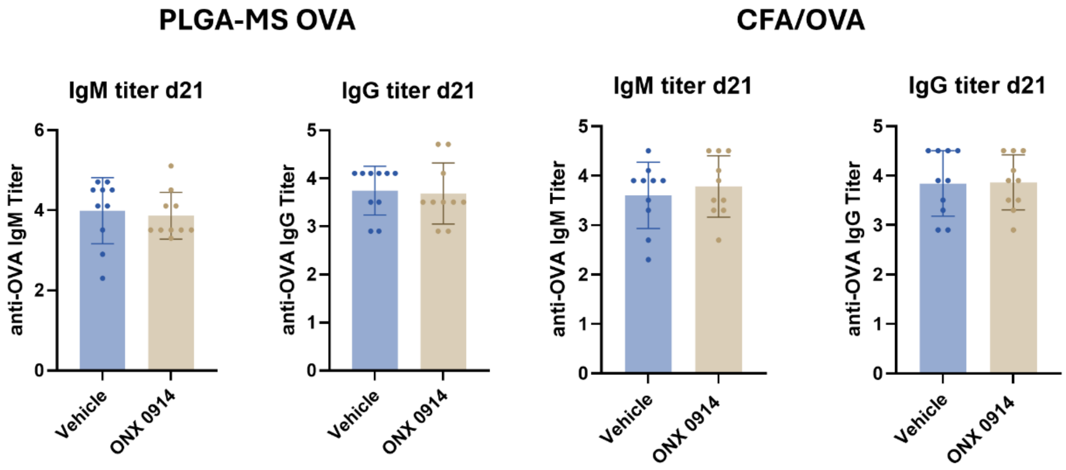

Supplementary Fig. 6: ONX 0914 treatment in two different OVA vaccination models does not impact serum anti-OVA antibody titers on day 21 after immunization. C57BL/6 or BALB/c mice (n=10 in each group) were s.c. immunized on day 0 with PLGA-MS OVA/Poly I:C or CFA/OVA, respectively. Starting one day prior to immunization, mice received 10 mg/kg ONX 0914 or vehicle (s.c.) 3x/week until sacrifice on day 28. Serum from immunized or naïve mice drawn on day 21 after immunization was analyzed via ELISA and anti-OVA IgM and IgG titers were determined. Antibody titers were defined as the decadic logarithm of the highest sample dilution exceeding the signal of the mean of naïve samples + 3x standard deviation (SD). Data is presented as means ± SD. Statistics: Student's t-test.

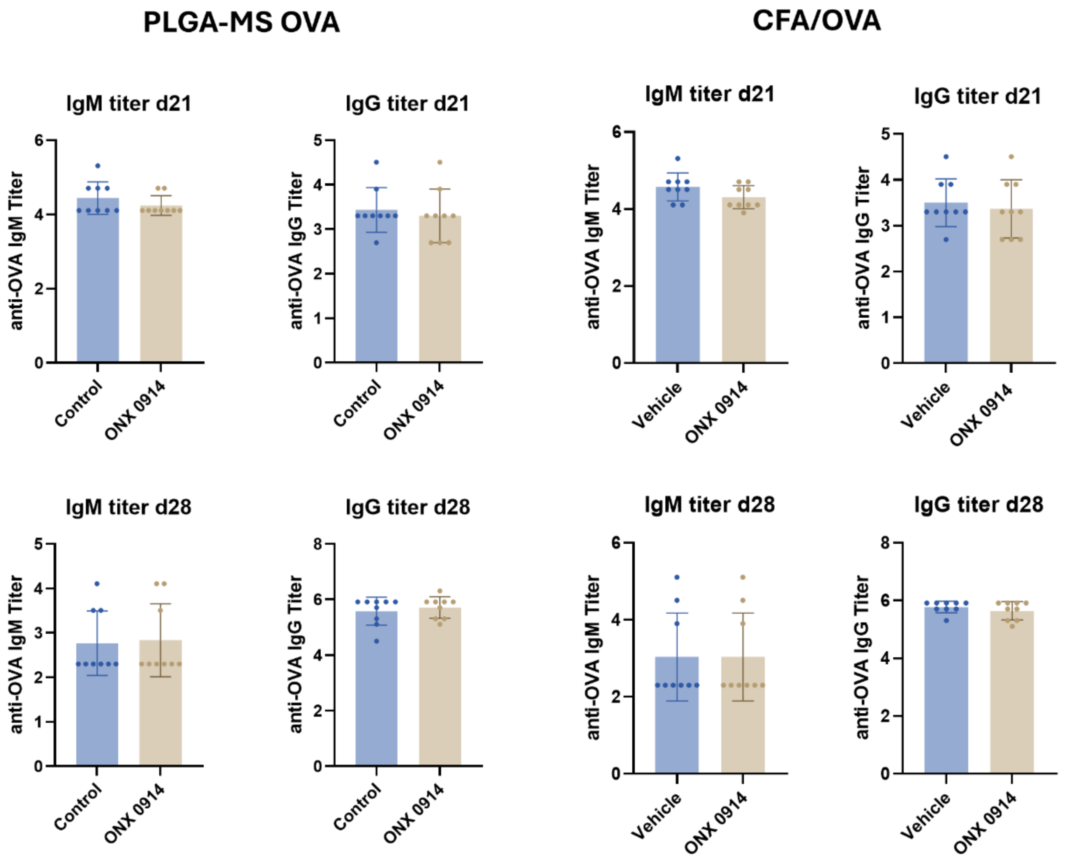

Supplementary Fig. 7: ONX 0914 treatment at later phases after OVA immunization does not impact serum anti-OVA antibody titers on day 21 after immunization. C57BL/6 or BALB/c mice (n=9 per group) were s.c. immunized on day 0 with PLGA-MS OVA/Poly I:C or CFA/OVA, respectively. 28 days post immunization, mice received 10 mg/kg ONX 0914 or vehicle (s.c.) 3x/week until sacrifice on day 35. Serum from immunized or naïve mice drawn on day 21 and 28 after immunization was analyzed via ELISA and anti-OVA IgM and IgG titers were determined. Antibody titers were defined as the decadic logarithm of the highest sample dilution exceeding the signal of the mean of naïve samples + 3x standard deviation (SD). Data is presented as means ± SD. Statistics: Student's t-test.

Supplementary Fig. 8: Uncropped Western Blots

Figure 1A

LMP2

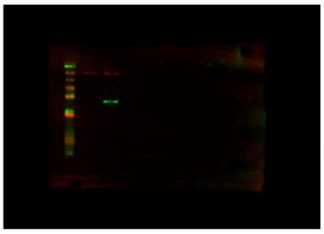

LMP7

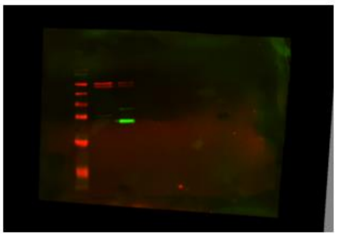

MECL-1

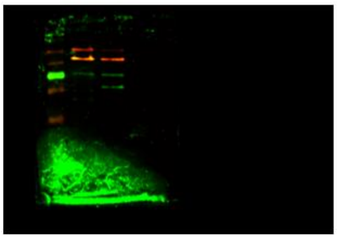

β1c

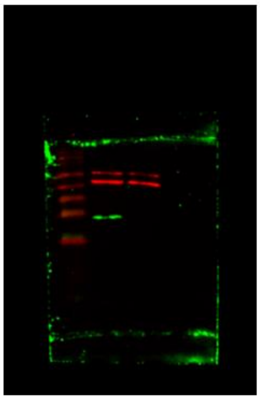

β2c

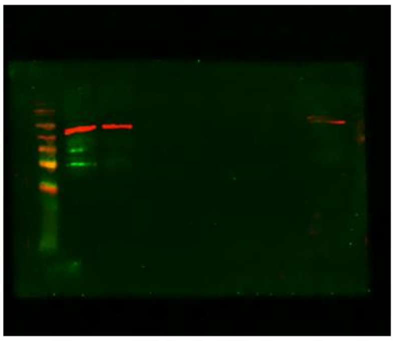

β5c

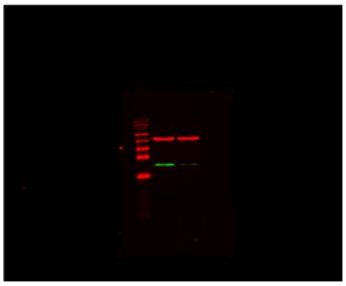

Figure 1c

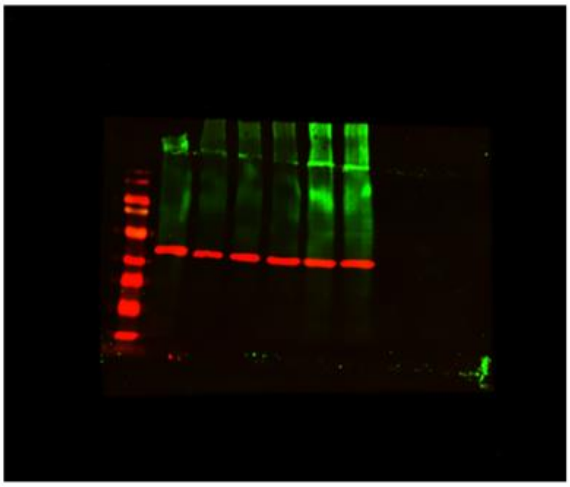

Supplementary Table 1: Primers for real-time RT-PCR

Name	Sequence	Supplier
sXBP-1 fwd	5'-CTG AGT CCG CAG CAG GTG-3'	Microsynth AG, Schweiz
s_uXBP-1 rev	5'-AGT TGT CCA GAA TGC CCA ACA-3	Microsynth AG, Schweiz
uXBP-1 fwd	5'-TCC GCA GCA CTC AGA CTA CG-3'	Microsynth AG, Schweiz
CHOP fwd	5'-AGA ACC AGG AAA CGG AAA CAG A-3'	Microsynth AG, Schweiz
CHOP rev	5'-TCT CCT TCA TGC GCT GCT TT-3'	Microsynth AG, Schweiz
ATF4 fwd	5'-GTT CTC CAG CGA CAA GGC TA-3'	Microsynth AG, Schweiz
ATF4 rev	5'-ATC CTG CTT GCT GTT GTT GG-3'	Microsynth AG, Schweiz
hRPL13a fwd	5'-GGA CCA TCG GCA TTT CT GTG-3'	Microsynth AG, Schweiz
hRPL13a rev	5'-AGT TTG GAG CGG TAC TCC TT-3'	Microsynth AG, Schweiz

Supplementary Table 2. List of antibodies for flow cytometry and western blot

Target	Clone	Specificity	Source	Catalog#	Used at
IgM	RMM-1	mouse	BioLegend	406506	1:200
CD19	6D5	mouse	BioLegend	115512	1:800
CD138	281-2	mouse	BioLegend	14508	1:400
BLIMP-1	5E7	mouse	BioLegend	150006	1:200
IgG2b	RMG2b-1	mouse	BioLegend	406703	1:500
IgG1	RMG1-1	mouse	BioLegend	406604	1:500
IgE	RME-1	mouse	BioLegend	406907	1:400
IgG	Poly-4053	mouse	BioLegend	405307	1:400
CD19	HIB19	human	BDBiosciences	555413	1:20
PSMB6 (β1)	E1K9O	human	Cell Signaling	13267S	1:1000
PSMB7 (β2)	E1L5H	human	Cell Signaling	13270S	1:1000
PSMB5 (β5)	D1H6B	human	Cell Signaling	12919S	1:1000
PSMB9(β1i)	E7J1L	human	Cell Signaling	87667S	1:1000
PSMB10 (β2i)	E6R7O	human	Cell Signaling	17579S	1:1000
PSMB8 (β5i)	D1K7X	human	Cell Signaling	13635S	1:1000
β-Actin	13E5	human	Cell Signaling	4970L	1:1000
γ-Tubulin	Polyclonal	human	Cell Signaling	5886S	1:1000
